# FDM 3D Printing and Properties of PBAT/PLA Blends

**DOI:** 10.3390/polym16081140

**Published:** 2024-04-18

**Authors:** Wangwang Yu, Mengya Li, Wen Lei, Yong Chen

**Affiliations:** 1 School of Mechanical Engineering, Nanjing Vocational University of Industry Technology, Nanjing 210023, China; 2 College of Science, Nanjing Forestry University, Nanjing 210037, China; 3 Jiangsu Province Precision Manufacturing Engineering and Technology Research Center, Nanjing 210023, China

**Keywords:** poly(lactic acid), polybutylene adipate terephthalate, blend, fused deposition molding, 3D printing, property

## Abstract

Biodegradable polylactic acid (PLA) has been widely used in fused deposition modeling (FDM) 3D printing. In order to improve its comprehensive properties in 3D printing, in this study, 0-40% content of polybutylene adipate terephthalate(PBAT) was selected to be blended with PLA in a twin-screw extruder; the resulting pellets were drawn into a homogeneous filament; then, PBAT/PLA samples were prepared by FDM 3D printing, and the effects of the dosage of PBAT on the mechanical properties, thermal behavior, surface wettability and melt flowability of the samples were investigated. The results showed that all the samples could be printed smoothly, and the ductility was slightly improved by the increase in the PBAT dosage; the thermal stability of PLA was enhanced by blending with PBAT, and the crystallinity increased monotonically with the increase in PBAT. After blending with PBAT, the surfaces of the samples were more hydrophilic and flowable. The important conclusion achieved in this work was that the PBAT/PLA blends, especially those containing 30%PBAT, showed great potential to replace petroleum-based plastics and are suitable for use in FDM 3D printing technologies for different applications.

## 1. Introduction

Fused deposition modeling (FDM), also known as fused filament fabrication (FFF), is one of the most rapidly growing additive manufacturing(AM) technologies [1,2,3,4]. The FDM printed products can be used in many areas, such as packaging supplies, automotive engineering, aeronautics, photocatalysis, bioengineering structures for tissue regeneration, dental implants, biomedical devices, and drug delivery [1,5,6].

In recent years, rising environmental awareness has made biodegradable polymers more attractive to scientific and industrial communities. As one of the two biopolymers that were expected to grow the most remarkably [7], PLA has become one of the routine feedstocks for FDM 3D printing; it is 100% obtained from renewable resources (corn, potatoes, tapioca roots, sugar cane, beets, maize, or rice); meanwhile, it has many advantages, such as adaptability to lower printing temperature, thermal stability, biodegradability, low thermal expansion coefficient, low elongation at break, slight shrinkage during processing, good adhesion to the platform, good dimensional stability of the printed specimens, ability to be carried out fast without any additional chemicals or biologically toxic materials, and it is mechanically robust [1,8]. Even so, its inherent drawbacks of high hardness, brittleness, poor melt strength, and no resistance to high temperatures should not be ignored; all these defects were unfavorable for its broader adoption in the 3D printing area. That was why many studies have devoted great attention to the modification of PLA for FDM 3D printing.

Recently, using the blends of various polymers for the FDM 3D printing technologies has aroused great interest from scientists and engineers. By using the blends, the printed samples usually occupied some unique properties when compared with that from a solo polymer; so, blending two polymers could be thought of as an efficient approach to more materials that are also suited for FDM 3D printing technology. For example, Davood et al. [5] found that PLA had a poor shape memory effect (SME) by itself, but this could be improved, obviously, by blending with TPU. The shape recovery ratio of the FDM printed TPU/PLA blend fell into the range between 90.9 and 96.4% under compression loading. Moreover, TPU droplets were found to be present in all PLA-TPU compounds, and an increased dosage of TPU would lead to larger continuous areas of TPU in the PLA matrix; the TPU phase was stretched after the FDM 3D printing. Among all the printing parameters, the infill density and nozzle temperature were found to have the greatest and least roles on the shape memory properties of the FDM 3D-printed TPU/PLA blend, whose weight composition was 30:70%, respectively [2]. Davood et al. [9] also investigated the SME of the FDM 3D-printed PVC/PCL blend, which was proved to have excellent SME, and the shape could be recovered by 100%. Liu et al. [10] constructed ABS/TPU/chlorella(ATCh40) and ABS/TPU (AT) skeletons using FDM technology, then integrated Fe_2_O_3_ onto the ATCh40(ATCh40-Fe_2_O_3_) and AT(AT-a-FeOOH), respectively. The photocatalysis behaviors of ATCh40-Fe_2_O_3_ and AT-a-FeOOH were comparatively investigated. They found that ATCh40-Fe_2_O_3_ exhibited a remarkable methyl orange removal rate of 91% within 240 min, while AT-a-FeOOH rate was only at 49%. This novel method, which used a blend of two polymers as skeletons, made it possible to develop new water treatment materials. Thavornyutikarn et al. [11] investigated the effect of the composition of ABS/TPU blends on the properties and printability of the extruded blend filaments; they noticed that the incorporation of TPU could improve the printability of the filament and decrease the warpage of the 3D-printed specimen. The blend containing 30% ABS and 70% TPU had the most homogeneous surface morphology; meanwhile, the printed ABS/TPU articles could absorb more energy when compared with those only from ABS. A commercial filament was used by Duigou et al. [12] for FDM printing; the filament was described as a blend of PLA and poly(hydroxyalkanoate) matrix reinforced with recycled wood fibers. The mechanical properties of the printed samples depended strongly on both the printing orientation and the printing width; the hygromorphism of the material enabled a shift toward 4D printing. Xu et al. [13] developed a new solvent mixing method to prepare the PLA/galactoglucomannan(GGM) blend, then extruded the blends into filaments for FDM printing scaffold prototypes; the printed objects could be applied in many areas, such as biomedical devices, owing that the versatile active sites in GGM may be used as carriers or molecular anchors to introduce desired functionality and features. Zhao et al. [14] developed various starch/PCL blends for low-temperature FDM; compared with those of PCL, the melting strength was enhanced, and the crystallization temperature, degree of crystallinity, and crystallization rate were increased when blending with starch; all these were beneficial for FDM process. Fekete et al. [15] probed the feasibility of the natural rubber(NR)/PLA blends for FDM printing, and the effect of the composition, the infill orientation on the quasi-static and dynamic mechanical properties, morphology, and thermal characteristics of the fabricated specimens were investigated. After investigation, they concluded that the excellent toughness made the NR/PLA filaments an eco-friendly substitution for ABS in 3D printing applications. NR was also chosen by Hassim et al. [16] to develop the filaments for FDM 3D printing by blending with ethylene–vinyl acetate (EVA). Though all the EVA/NR blends were unsuccessfully printed due to buckling attributed to the material behavior, different thermoplastic and nano/bio fillers were thought to be able to improve printability and provide desirable properties suitable for a specific application when they were involved in the ingredients.

PBAT is a biodegradable aliphatic polyester that is fully degradable under composting conditions and has excellent ductility and thermal stability [17]; there exist some complementarities between the physical–mechanical properties of PLA and PBAT; therefore, some researchers have focused on the development of PBAT/PLA blends by traditional processing methods. For example, Barbosa et al. [18] prepared two kinds of PBAT/PLA blends by extrusion and injection molding. For the first blend, both maleic anhydride(MA) and dicumyl peroxide were used as additives; for the second one, no additives were adopted. They found that the application of the additives promoted better interactions between the two phases, and the blend, thus, had better mechanical and thermal behaviors; the two blends had much higher impact resistance values than pristine PLA, showing the role of the PBAT to improve the toughness. Schmitz et al. [19] melt blended PBAT and PLA using a twin-screw extruder and then injected into the specimens for mechanical tests and compression molded into plates for the biodegradation study, respectively. In the blend, poly(styrene-co-maleic anhydride) was chosen as a compatibilizer. The impact strength was increased significantly from 27 J/m for pure PLA to 109 J/m for the blend with the addition of 80 wt% of PBAT; meanwhile, the elongation at break was from 2.2 to 35%, and the toughness of PLA was obviously enhanced. The adoption of PBAT in the blend was helpful for the degradation of PLA in a simulated marine environment. In order to improve the compatibility between PBAT and PLA, an epoxidized cardanol-based prepolymer (ECP) generated from cashew nut shell liquid and a petroleum-derived glycidyl-based copolymer, namely, Joncryl ADR4300 (ADR) (molar mass = 5500 g mol^−1^; epoxide equivalent = 445 g mol^−1^; glass transition temperature (T_g_) = 56 °C, produced by BASF, Ludwigshafen, Germany), were chosen by Silva et al. [20] as the compatibilizers in the PBAT/PLA blends. PLA, PBAT, and ADR or ECP were dry-mixed and melt-blended using a torque rheometer. Then, triphenyl–phosphine was introduced into the blend as a catalyst. After blending, the materials were milled and injection molded. After investigation, they concluded that the addition of ECP resulted in significant improvement in the mechanical properties of the blend, which were similar to or even superior to those observed for ADR. Moreover, a sea-island-type morphology with very small PBAT domains in the blend could be observed. ECP could be considered a cost-effective and environmentally-friendly biobased compatibilizing agent for the PBAT/PLA blends.

Motivated by the aforementioned ideas, blending with PBAT should be an effective method to improve some properties of PLA. In order to make clear the possibility of the PBAT/PLA blend as a candidate for AM technology, in this study, PLA was blended with PBAT in different ratios and FDM 3D printed to obtain a series of printed specimens. At the same time, the FDM 3D-printed PLAs were also prepared for comparison. The mechanical properties, morphology, thermal stability, melting and crystallization behavior, hydrophilicity, as well as mobility of the samples were characterized. This research aims to interpret the advantages and disadvantages associated with the PBAT/PLA blends and explore the potential use of the blends as filaments for FDM 3D-printing technology. The main problem to be solved in this study is to make clear the optimal composition of the blend, which not only has the best comprehensive physico-mechanical properties, but also can be printed smoothly.

## 2. Materials and Methods

### 2.1. Materials

PLA 3052D in ellipsoidal pellet forms was purchased from Suzhou Benfuzhong Plastic Import and Export Co., Ltd., China (Suzhou, China); its glass transition temperature (T_g_) is between 55 °C and 60 °C, and its average sizes of the elliptical major and minor axes are 3.82 mm and 3.08 mm, respectively. PBAT TH803S in ellipsoidal pellet forms was purchased from Xinjiang Blue Ridge Tunhe Sci. & Tech. Co., Ltd., China (Changji, Xinjiang, China); its glass transition temperature (T_g_) is −29.3 °C, and its average sizes of the elliptical major and minor axes are 4.77 mm and 1.73 mm, respectively.

### 2.2. Sample Preparation

Prior to blending, the PLA and PBAT underwent drying in a blast oven at temperatures of 60 °C to constant masses to remove the adsorbed moisture. Subsequently, the two polymer materials were accurately weighed according to the ingredients listed in Table 1 and uniformly mixed; then, they were melt-blended and pelletized utilizing a twin-screw extruder (SHJ-20, Nanjing Giant Machinery Co., Ltd., Nanjing, China); the diameter of its screw was 21.7 mm; aspect ratio was 40:1; output was 1–5 kg/h; the temperatures from zone I to zone VI and the head were 155 °C, 160 °C, 165 °C, 175 °C, 170 °C, 165 °C, and 160 °C, respectively; the rotating speed of the screw was 70 r/min.

**Figure 1:**
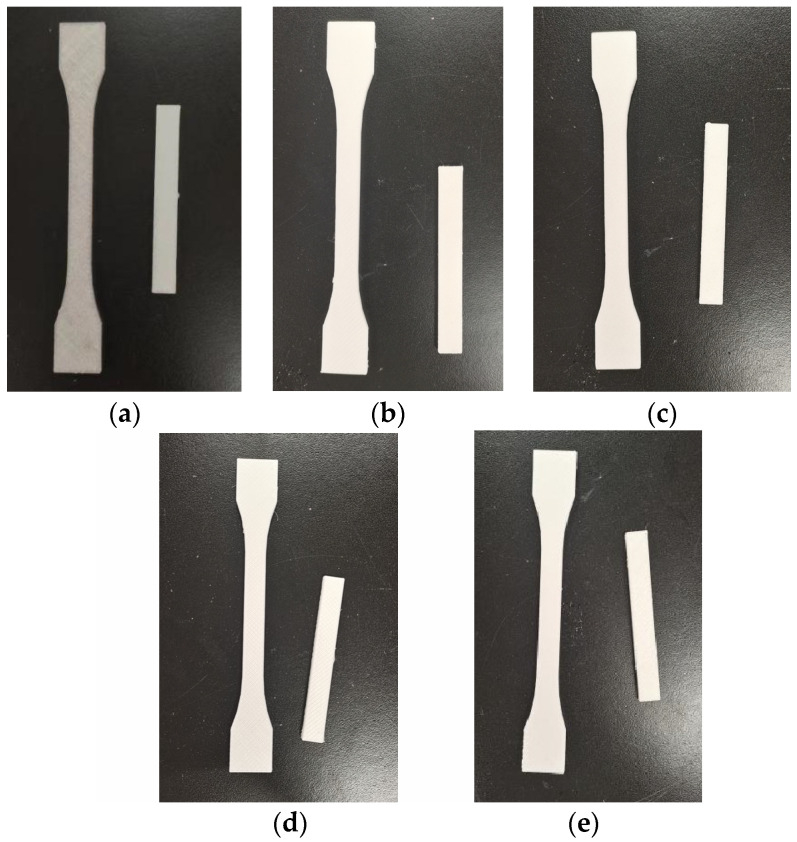
Tensile and flexural test specimens: (**a**) PLA; (**b**) 10% PBAT/PLA; (**c**) 20% PBAT/PLA; (**d**) 30% PBAT/PLA; (**e**) 40% PBAT/PLA.

The resulting pellets from the above granulator were subsequently added into a single-screw extruder (KS-HXY, HUANXINYANG Electrical Equipment Co., Ltd., Suzhou, China) at 155 °C and a rotating speed of 20 rpm to produce filaments for printing, and the diameter of the filament was controlled within 1.75 ± 0.05 mm. The desktop 3D printer employed for this study was the MOSHU S108 model (Hangzhou SHINING 3D Technology Co., Ltd., Hangzhou, China); the test specimens were printed using the optimal printing parameters reported in our previous work [21]: a nozzle temperature of 220 °C; a platform temperature of 50 °C; a printing speed of 50 mm/s; a layer thickness of 0.1 mm; and an infill density of 100%. For example, the printed samples for tensile and flexural tests are shown in Figure 1.

### 2.3. Testing and Characterization

#### 2.3.1. Mechanical Testing

The specimens for the tensile and flexural tests were made according to the ASTM D 638-2010 [22] and the ASTM D 790-2010 [23], respectively; the tensile properties were tested according to the mentioned standard at a cross-head speed of 10 mm/min and the flexural properties at a cross-head speed of 5 mm/min, using a universal mechanical testing machine (E44.304, MTS Industrial Systems (China) Co., Ltd., Shenzhen, China) with a 20 kN load capacity.

#### 2.3.2. Morphology Observation

The morphology of the fractured surface of the specimens during the tensile test was obtained using a field-emission scanning electron microscope (SEM) (Hitachi SU 8010, Hitachi Corporation, Tokyo, Japan) at an accelerating voltage of 3 kV. For a better resolution, the fracture surface of each sample was coated with a thin layer of gold before the SEM examination to improve the surface conductivity.

#### 2.3.3. Thermal Stability Assessment

The thermal stability of the specimen was assessed by the thermo-gravimetric analysis (TGA) technology. A thermo-gravimetric analyzer (NETZSCH-Gerätebau GmbH, Selb, Germany) was employed to carry out the examination using nitrogen as the purge and protective gas, whose flow rate was 20 mL/min. The sample mass was from 6 to 8 mg; the heating rate was 20 °C/min, and the temperature range was from 20 °C to 600 °C. The initial thermal decomposition temperature (T_i_) and the maximum degradation temperature(T_p_) at which the sample lost its weight the most, corresponding to the peak temperature in the first-order derivative weight curve, and the percentage char (w%) were observed.

#### 2.3.4. Melt and Crystallization Behavior Determination

The crystallization and melting behaviors of the specimens were evaluated using a differential scanning calorimeter (DSC) (DSC214, NETZSCH-Gerätebau GmbH, Selb, Germany). The 5–10 mg samples were heated at 15 °C/min from 20 to 220 °C under nitrogen and stayed at this temperature for 15 min to erase the thermal history, residual moisture, and voids. Then, the samples were cooled down to room temperature and heated again to 220 °C at the same heating rate. The transition temperatures and heat capacities were calculated using the NETZSCH analysis software. The crystallinity was calculated according to the following equation, considering the polymer fraction in the printed sample:

(1) $$x_{c}(\%)=\frac{\left|\Delta H_{m}+\Delta H_{cc}\right|}{\phi \Delta H^{\theta }}\times 100$$

where $x_{c}$ represented the degree of crystallinity of the sample; ϕ was the actual weight fraction of PLA in the blend; $\Delta H_{m}$ was the experimental melt enthalpy (J/g); $\Delta H_{cc}$ was the experimental cold crystallization enthalpy (J/g), and $\Delta H^{\theta }$ was the theoretical enthalpy value for 100% crystalline structure, which was 93 J/g according to the literature [8,24].

#### 2.3.5. Water Contact Angle Measurement

The contact angle of distilled water drop on the surface of each FDM 3D-printed specimen was measured at room temperature using a contact angle instrument (DSA100; KRÜSS GmbH, Borsteler Chaussee, Hamburg, Germany). A 5 µL droplet of distilled water was dropped onto the surface and kept for 15 s, and then the contact angles from the images were measured at different points.

#### 2.3.6. Melt Flow Index Measurement

The melt flow index (MFI) of the samples was examined based on Chinese National Standard GB/T 3682-2000 [25]. The samples were weighed and put into the melt index meter (XNR-400, Chengde Jinhe Instrument Manufacturing Co., Ltd., Chengde, China). The MFI measurements were carried out at defined temperatures and 2.16 kgf. The interval between cutting sections was 30 s, and the reference time was 10 min.

## 3. Results and Discussion

### 3.1. Mechanical Properties

**Figure 2:**
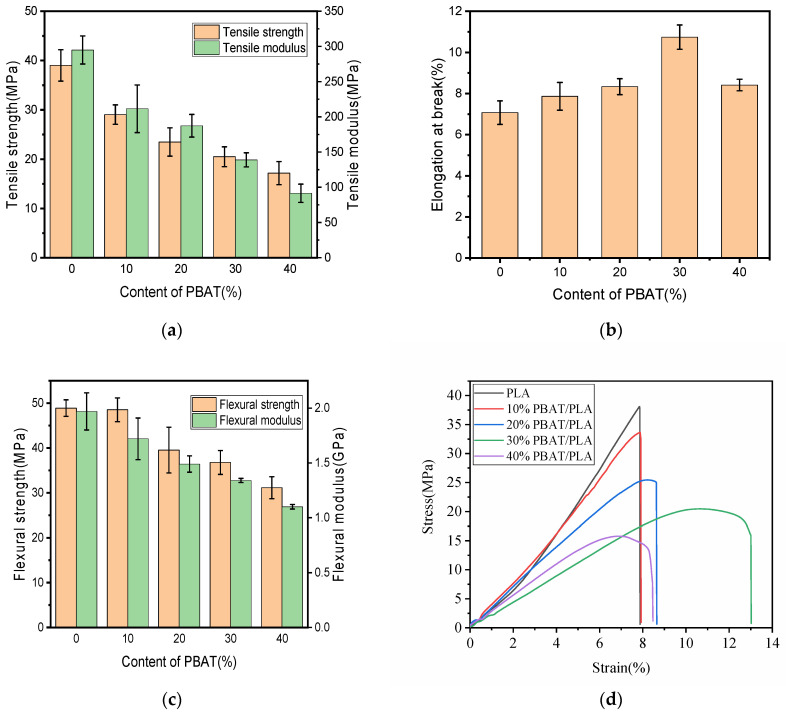
Mechanical properties of PBAT/PLA with different contents of PBAT: (**a**) tensile properties; (**b**) elongation at break; (**c**) flexural properties; (**d**) stress–strain curve.

Mechanical properties are usually very important for the performance evaluation of 3D-printed objects. The tensile and flexural properties of FDM 3D-printed PLA and PBAT/PLA blends are depicted in Figure 2. According to the findings, the mechanical properties of the blends altered drastically with the variation in PBAT content.

From Figure 2a, the obtained PLA sample had a tensile strength of 39.01 MPa, which was quite close to the value of 42.27 MPa of the previous study [26]. After being blended with PBAT, the tensile strength of PLA would be reduced obviously, and this reduction became heavier when more PBAT was incorporated into the 3D-printed blend specimens; this decrease in tensile strength while increasing PBAT content had also been evidenced in other studies concerning injection molded PBAT/PLA bends [27,28]. This may be due to the poor compatibility between the two polymer phases. The tensile modulus in Figure 2a showed a similar changing trend with the tensile strength. When 40% PLA was substituted with PBAT, the tensile strength and modulus of the printed specimen reduced to 17.16 MPa by 56.01% and 91.47 MPa by 69.00% from those of neat PLA, respectively.

Figure 2b shows the flexural properties of the 3D-printed samples; it could be found that both the flexural strength and modulus behaved similarly with the tensile properties; they both reduced gradually with the increasing content of PBAT; for the 40% PBAT/PLA sample, its flexural strength and modulus were only 31.13 MPa and 1.09 GPa, dropping greatly from those of neat PLA, which were, correspondingly, 48.90 MPa and 1.97 GPa.

Despite the fact that both the tensile and the flexural strengths became poorer when more PBAT was incorporated, the variation in the flexural strength with the load of PBAT behaved a little differently from that of the tensile strength; for the former, it only dropped slightly by 0.76% when 10% PBAT was used, and after that, it would reduce quickly, while for the tensile strength, it would be decreased remarkably by 30.33% when 10% PBAT was used; then the decrease became much slower when more PBAT was adopted; this difference might arise from the different fracture mechanisms between them; the tensile samples failed mainly because of the tensile force in the middle, while the load in a three-point bending test was the combination of the compressive force at the top, the shear force in the middle, and the tensile force at the bottom [29].

Theoretically, the ductility of PLA should be improved after being blended with PBAT thanks to the good flexibility of PBAT, as seen in Figure 2b. However, the improvement in the elongation at break (EAB) of the sample increased but not significantly; the EAB of neat PLA was 7.07%. This result has also been observed by some other researchers [8,30]. After being blended with PBAT, the EAB value of the sample rose slightly, and even for the 30% PBAT/PLA, which had the greatest EAB among all the blends, its value was only 10.15%. The little variation in EAB meant that the sample, after blending, would still break in a brittle manner, which might be because of the poorer interface bonding between the two polymers in the blends. The typical stress–strain curves shown in Figure 2d also demonstrated that PLA and the samples containing less PBAT broke in a brittle manner; when 30 wt% or 40 wt% PBAT was used, a yield point appeared in the curve, but not obviously; in this situation, the ductility of the sample was slightly enhanced but generally broke in a brittle manner.

Mechanical properties of polymers are always relevant to their applications, and from the above discussion, it was learned that blending with PBAT worsened the tensile and flexural strengths and moduli of PLA, but the absolute values were still relatively high; even for the 40%PBAT/PLA specimens, they still had greater strengths and moduli than some bio-polymers, such as starch plastics and PCL [31]. In summary, the printed PBAT/PLA blends were suitable for many actual applications.

### 3.2. Morphology

**Figure 3:**
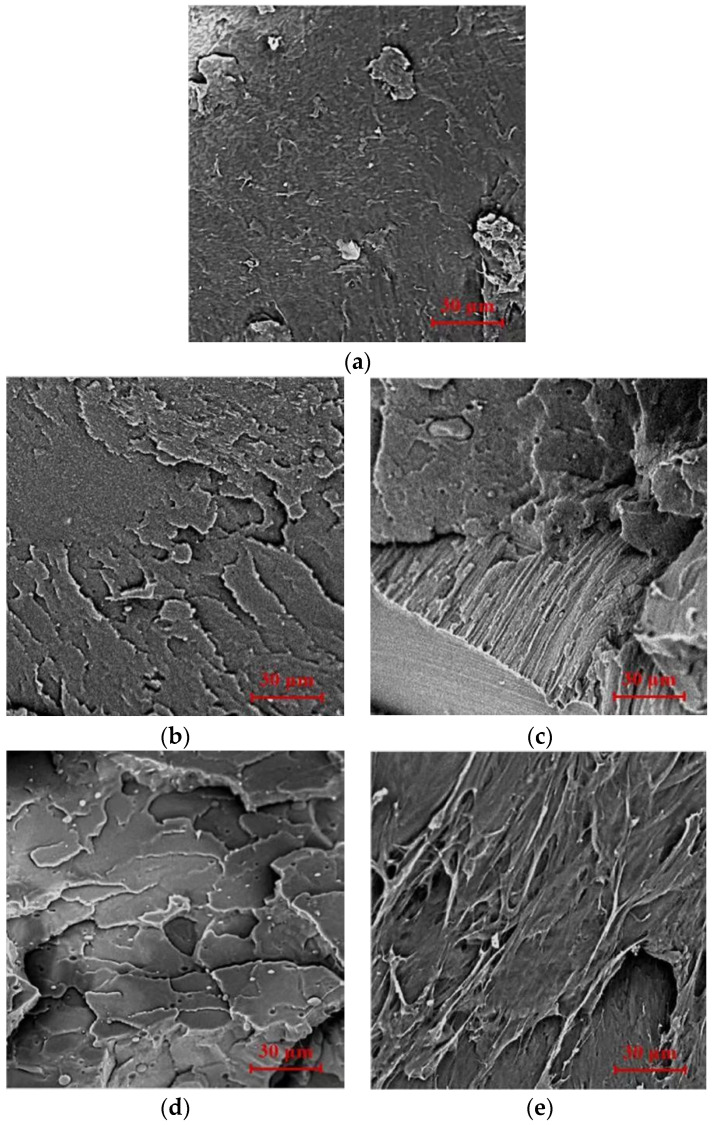
SEM Images of neat PLA and various PBAT/PLA blends with a magnification of 2000 times: (**a**) PLA; (**b**) 10% PBAT/PLA; (**c**) 20% PBAT/PLA; (**d**) 30%PBAT/PLA; (**e**) 40% PBAT/PLA.

Figure 3 displays the scanning electron microscopy (SEM) images of the breaking surfaces of the printed specimens containing various contents of PBAT. These images provide essential information about each specimen’s microstructural characteristics and fracture behavior.

The fracture surface of pristine PLA was smooth and flat, as observed in the SEM images (Figure 3a), indicating that PLA broke in a brittle manner; this finding was consistent with earlier studies [32]. The fracture surfaces of the 10% PBAT/PLA and 20% PBAT/PLA printed specimens shown in Figure 3b,c looked like that of PLA, indicating that blending PLA with a small amount of PBAT would not change the fracture behavior of the printed samples. When more PBAT was adopted, the fracture morphologies of the 30% PBAT/PLA and 40% PBAT/PLA printed specimens became a little rougher (Figure 3d,e), revealing that the ductility of the blends would be improved. However, the change in the morphology was so little that the samples would become brittle when fractured, which was consistent with the EAB results shown in Figure 2b and the stress–strain curves shown in Figure 2d.

### 3.3. Thermal Stability

**Figure 4:**
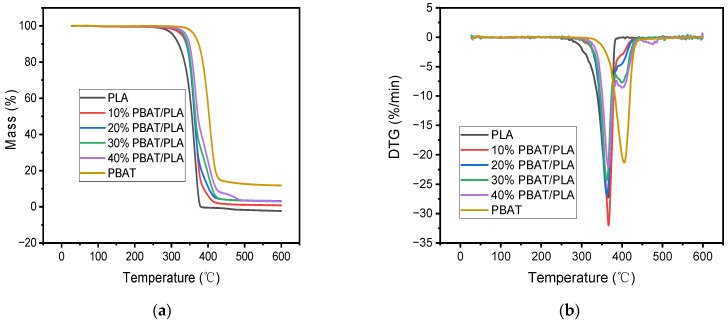
Pyrolysis process of different samples: (**a**) mass loss curve; (**b**) differential thermogravimetric curve.

Thermal stability was another critical parameter besides the mechanical properties used to evaluate the performance of 3D printing materials; that is why many literature sources have reported on it [13,26,33]. The thermal stability of PLA and PBAT/PLA blends was investigated using TGA analysis; the percentage weight change and the derivative of weight as a function of each material’s temperature are illustrated in Figure 4. For comparison, the thermal properties of PBAT granules were also investigated. From Figure 4, it was clear that the PBAT began to decompose at a much higher temperature than the neat PLA; its maximum degradation temperature at which the sample lost its weight at the greatest speed corresponding to the peak temperature in its DTG curve and char residue were both greater than the latter, showing that PBAT was more thermally stable than PLA. Additionally, both plastics deteriorated in one step. In the case of PBAT/PLA blends, however, the addition of PBAT had a great influence on the thermal degradation process of PLA. Firstly, increasing the dosage of PBAT reduced the biocomposite char residue; secondly, the T_i_ value became greater than that of PLA, which might be attributed to the more thermal stability of PBAT than PLA as aforementioned, and the value increased continuously with increasing PBAT content in the blend, meaning that the introduction of PBAT improved the thermal stability of PLA, and a greater dosage of PBAT made the blend more thermally stable; thirdly, the thermal decomposition of each blend was finished in two steps, and two distinct peaks, T_p,1_ and T_p,2_, could be found in the DTG curve of each blended specimen, which was quite different from those of neat PLA and PBAT. The two peaks might correspond to the thermal decomposition of PLA and PBAT. Generally, the second peak at a higher temperature (T_p,2_) in the blend containing more PBAT became more distinct, indicating that the phase separation between the two polymers became more serious, and the interfacial compatibility and cohesion between PLA and PBAT became poorer.

**Figure 5:**
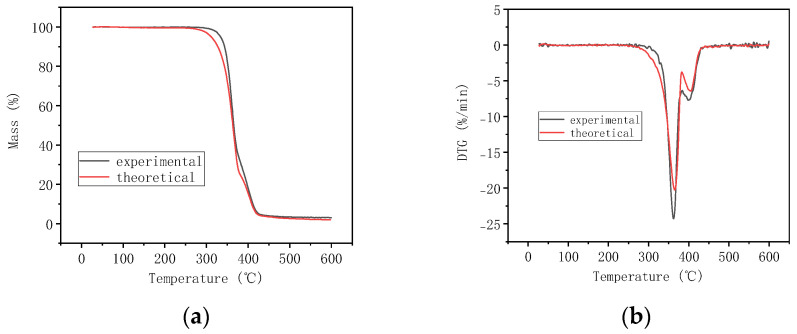
Calculated and experimental curves of 30% PBAT/PLA: (**a**) mass loss curve; (**b**) differential thermogravimetric curve.

In order to indicate some reaction of the interaction between the two matrices, the TGA data of 30% PBAT/PLA as an example were theoretically calculated from those of PLA and PBAT; the calculated curves and the experimental curves were demonstrated simultaneously in Figure 5.

From Figure 5, it can be seen that the theoretical curve has a lower T_i_ value than that of the experimental curves. The results implied that the blend was more thermally stable than the simple mixture of PBAT and PLA, revealing some interaction between the two polymers when they were blended.

Moreover, we once investigated the thermal stability of FDM 3D-printed PBS/PLA samples and found that the thermal stability of the PBS/PLA blends would be worsened when more dosage of PBS was incorporated [34]. The improved thermal stability of FDM 3D-printed PBAT/PLA blends indicated that the PBS/PLA blends can be replaced with the PBAT/PLA blends when the thermal stability of the samples is taken into consideration. In other words, the FDM 3D-printed PBAT/PLA blends can be applied at higher temperatures than the PBS/PLA blends.

### 3.4. Melt and Crystallization Behavior by DSC

**Figure 6:**
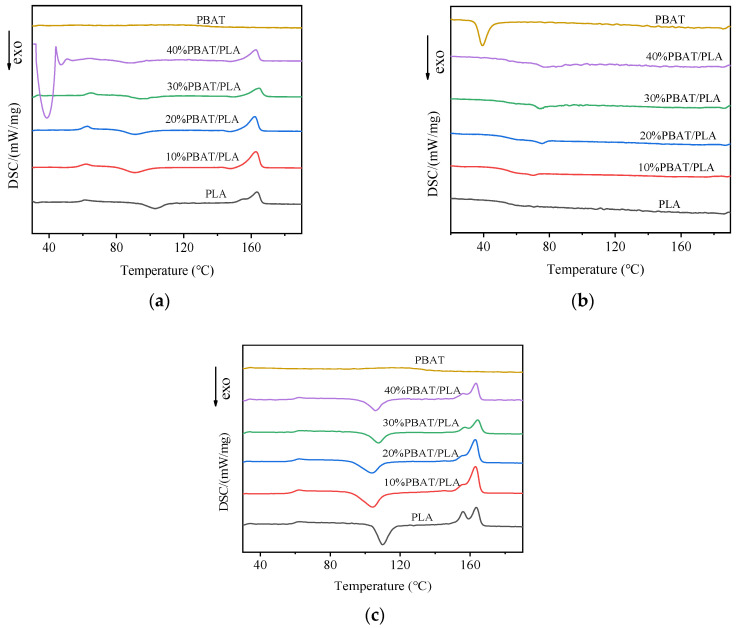
DSC thermograms of printed samples with different contents of PBAT: (**a**) first heating; (**b**) cooling; (**c**) second heating.

DSC tests were often employed to characterize the melt and crystallization characteristics of polymers and their composites [24]. Here, the test was performed by submitting the materials to two heating scans interspersed by a scan at a controlled cooling speed. The results of the DSC test on PLA, PBAT, and the PBAT/PLA blends are reported in Figure 6. The corresponding DSC characteristics data are tabulated in Table 2.

Based on the cooling curves in Figure 6b, no clear melting–crystallization peak could be found for neat PLA and all the blends. However, a big cold-crystallization peak appeared during the second heating process. As shown in Figure 6c, the crystallization peaks were all greater, and the temperatures at the peaks were also higher than those that happened during the first heating cycle (Figure 6a); in the second heating cycle, the cold crystallization (T_cc_) took place at 109.9 °C for neat PLA and between 104.0 °C and 107.6 °C for the PBAT/PLA blends. In comparison to neat PLA, the T_cc_ values of PBAT/PLA blends have decreased somewhat with the inclusion of PBAT, indicating that the incorporation of PBAT could improve the crystallization behavior of PLA, which might be due to the nucleating action of PBAT as the secondary polymeric phase on the crystallization of PLA because of their imperfect miscibility [24]. The fact that the nucleation of the PLA could be increased in the presence of the second polymer inside the PLA has also been found by Chuayjuljit et al. [35].

The calculated degree of crystallinity in Table 2 provides evidence of these phenomena; the X_c_ values of all the blends were greater than that of neat PLA, and the X_c_ value of 40%PBAT/PLA was even 3.11 times that of neat PLA. In addition, a distinct shoulder melting peak emerged at 155.6 °C beside its main melting peak at 163.3 °C for neat PLA in the second heating plots (Figure 6c), suggesting the formation of meta-stable and perfected crystals [36]. Nevertheless, after being blended with PBAT, the exothermic peak of crystallization at the lower temperature almost faded, proving that the crystallization of PLA became more perfect in this situation.

When the glass transition temperature (T_g_) is concerned, it can be found in Table 2 that PLA had a T_g_ of 59.9 °C, almost the same as the previous reports [37]. Table 2 also lists that there are no major differences in the T_g_ values of the blends and PLA; in other words, it can be concluded that the addition of PBAT has very little effect on the glass transition temperatures of PLA. This may be because of the poor interfacial compatibility in the blend, leading to the little influence of PBAT on the mobility of the PLA molecular chains.

### 3.5. Wettability

**Figure 7:**
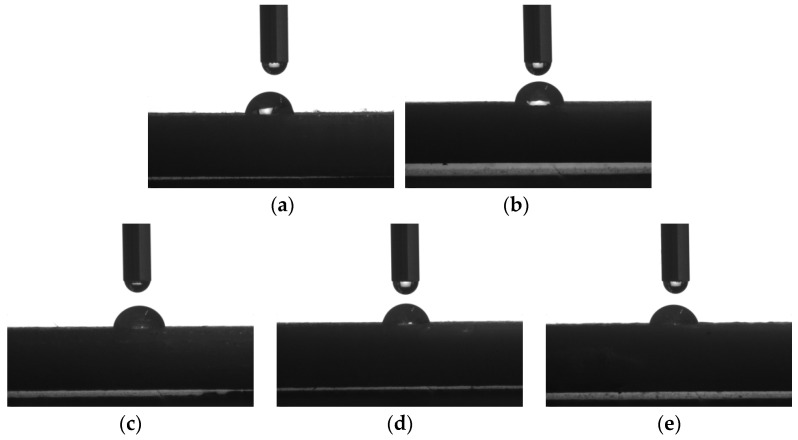
Contact angle images of distilled water on the surfaces of printed samples: (**a**) PLA; (**b**) 10% PBAT/PLA; (**c**) 20% PBAT/PLA; (**d**) 30%PBAT/PLA; (**e**) 40% PBAT/PLA.

The wettability, i.e., the surface hydrophilicity of the printed sample, could be indicated by the water contact angle on its surface, which was formed by the intersection of the water-printed sample interface and the water–gas interface. The shapes of the water droplets on the surfaces of the printed samples are presented in Figure 7. Table 3 tabulates the measured average contact angles of the samples.

It can be noticed from Figure 7 and Table 3 that the contact angles of all the samples are smaller than 90°, indicating that the surfaces of all the samples are hydrophilic and could be wetted with water; furthermore, the water contact angles for the specimens decreased monotonically with the increasing dosage of PBAT; the contact angle on the surface of PLA was 82.96° when 10%, 20%, 30%, and 40% PBAT was used; the contact angle reduced by 2.8°, 5.32°, 6.9°, and 9.12°, respectively. The water contact angle results demonstrated that PLA could be integrally hydrophilically modified by PBAT.

Nowadays, more and more natural fibers are incorporated into polymer complexes to prepare the feedstocks for FDM 3D printing [26,38]; the interfacial compatibility between the fiber and the polymer matrix is key for the properties of the printed samples. Being mainly composed of cellulose, hemicellulose, and lignin, the natural fiber has many hydrophilic groups on its molecular structure [4,29]. When the hydrophilicity of PLA is enhanced with PBAT, the mutual miscibility between natural fiber and the PBAT/PLA blend will become better, and consequently, the comprehensive performances of the printed samples can be improved. The improved hydrophilicity of the blend shown in Table 3 implies that more biocomposites based on natural fiber and the PBAT/PLA blend can be developed for FDM 3D printing.

### 3.6. Melt Flow Index

**Figure 8:**
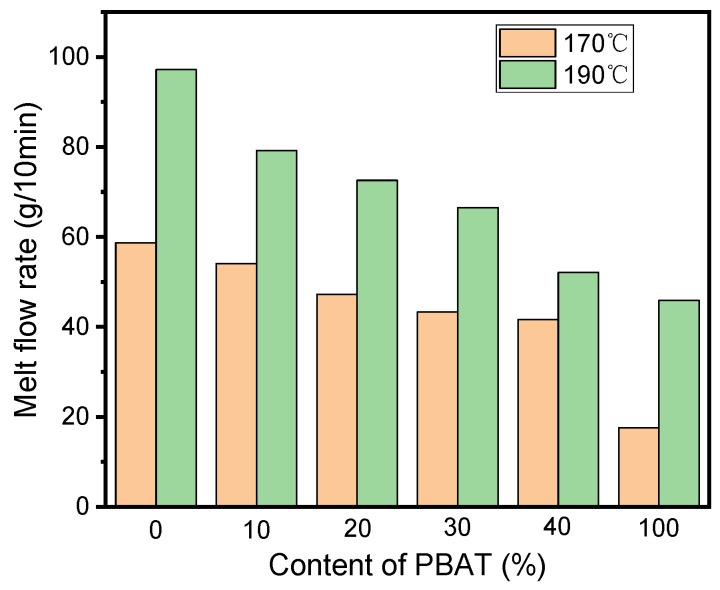
Melt flow index curves of different samples.

The rheological properties of the materials are very important for the FDM 3D printing process; proper rheological behavior is helpful for the easy extrusion of the melt through the nozzle and can avoid shape instability and defect formation of the product effectively. Generally, the rheological behavior of a polymer material can be obtained using a rheometer; some key factors affecting the flowability of the melt, such as the complex viscosity as well as the relationship between the viscosity and the shear rate, can be obtained intuitively [39,40]. As a simplification method, the processability of a polymer material for 3D printing could also be represented with its MFI [41], which was directly dependent on the structural and molecular arrangement of the polymer [42] and can be used to describe the uniformity of the flow rate of thermoplastic materials [43]. For the FDM process, the MFI of a polymer was not only important in defining the printing parameters, but it also affected the bonding between printed layers. Therefore, investigating this critical manufacturing feature was essential for the printing process and the final quality. Figure 8 illustrates the melt flow rate findings of the PLA and PBAT matrices and the blends with different PBAT loads.

It was noted that the MFI values of all the blends were greater than that of PBAT while lower than that of PLA; the MFI value exhibited almost a linear decrease from the highest to the lowest with the increasing PBAT load in the blends at the two experimental temperatures of 170 °C and 190 °C. The relationship between the content of PBAT(X) in the blend and the MFI value (Y) of the printed samples at 170 °C and 190 °C could be expressed by the regression equations Y = 58.78 − 0.448X (R^2^ = 0.9614) and Y = 94.1 − 1.029X (R^2^ = 0.9609), respectively. On the basis of the obtained equations, it was found that the PBAT load had a great effect on the flow ability of the melt, and this effect was much heavier at the higher processing temperature, which could be discerned from the greater slop of the line for 190 °C than that for 170 °C. The gradual decrease in the MFI value of the blend with the increasing dosage of PBAT might have resulted from the much lower MFI value of PBAT than that of PLA, characterizing the resistance of PBAT to the flow of PLA melts. Moreover, PBAT might act as the nucleating agent in the blends and increase the crystallinity of PLA, leading to lower MFI values. The correlation of the increased crystallinity of the polymer with the decreased MFI value has also been reported by Kaczor et al. [44]. In our study, all these findings were validated in the thermal stability and the DSC and XRD analyses sections. It is worth noting that, even for 40%PBAT/PLA, its MFI was still high enough for FDM 3D printing, though it was the lowest in all the blends.

## 4. Conclusions

PLA and PBAT were melt-blended on a twin-screw extruder and pelletized; then filaments were prepared from the pellets using a single screw extruder, and finally, a series of PLA/PBAT blends containing 0–40 wt% PBAT were FDM 3D-printed. The following conclusions can be drawn:

1. PBAT incorporation into PLA could improve the flexibility and ductility of PLA, but not significantly, and also at the expense of reducing tensile and flexural strength and stiffness. The highest elongation at break was obtained for the 30% PBAT/PLA blend, but even so, it was only 10.15%. All the printed samples would fracture mainly in a brittle manner. The results of the morphology characterization were consistent with the mechanical properties, and the mixing of PBAT changed the fracture morphology from smooth and flat to rough, but only slightly;
2. The addition of PBAT was found to improve the thermal stability of the PLA, and the crystallinity increased monotonically with the PBAT content; the crystallinity of the 30% PBAT/PLA sample increased from that of PLA by 142.97%;
3. The hydrophilicity of the blend was enhanced when more PBAT content was used, which was favorable for compounding with natural fibers.

Although increasing the dosage of PBAT would reduce the MFI of the blend, its MFI was still sufficient for FDM 3D printing, even if the PBAT dosage was as high as 40%.

To sum up, the interfacial compatibility in the PBAT/PLA blend was not so good, but blending with PBAT contributed to improving the comprehensive printing performance of PLA, in which the optimal doping amount of PBAT was 30%. In the future, more ways should be found to improve the interfacial bonding; meanwhile, the PBAT/PLA blends could be complexed with some natural fibers to develop more filaments for FDM 3D printing.

## Figures and Tables

**Table 1:** Nomenclature of printed samples based on weight fractions of different components.

Sample Codes	PLA	10% PBAT/PLA	20% PBAT/PLA	30% PBAT/PLA	40% PBAT/PLA
PLA/wt%	100	90	80	70	60
PBAT/wt%	0	10	20	30	40

**Table 2:** The DSC testing outcomes of PLA, PBAT, and their blends.

Sample Codes	T_g_/°C	T_cc_/°C	∆H_c_ (J/g)	∆H_m_ (J/g)	X_c_ (%)
PLA	59.9	109.9	25.49	26.69	1.28
10% PBAT/PLA	59.8	104.1	32.05	33.54	1.77
20% PBAT/PLA	59.5	104.0	23.92	25.47	2.07
30% PBAT/PLA	61.4	107.6	17.50	19.54	3.11
40% PBAT/PLA	60.3	105.9	15.54	17.78	3.98
PBAT	/	/	/	8.252	7.23

**Table 3:** Contact angles of distilled water on the surfaces of printed PLA and PBAT/PLA blends.

Sample Codes	PLA	10% PBAT/PLA	20% PBAT/PLA	30% PBAT/PLA	40% PBAT/PLA
Contact angle/°	82.96	79.16	77.64	76.06	73.84

## Data Availability

Data are contained within the article..
